# Molecular Investigation of *Eimeria* spp. Infection in Weaned Dairy Calves in Thessaly, Greece, and Associated Risk Factors

**DOI:** 10.3390/ijms27062903

**Published:** 2026-03-23

**Authors:** Konstantinos V. Arsenopoulos, Sotiris Chrysanthopoulos, Elias Papadopoulos

**Affiliations:** 1 Department of Veterinary Medicine, School of Veterinary Medicine, University of Nicosia, Engomi, 2414 Nicosia, Cyprus; chrysanthopoulos.s@live.unic.ac.cy; 2 Laboratory of Parasitology and Parasitic Diseases, School of Veterinary Medicine, Faculty of Health Sciences, Aristotle University of Thessaloniki, 54124 Thessaloniki, Greece; eliaspap@vet.auth.gr

**Keywords:** *Eimeria* spp., dairy calves, weaning, multiplex PCR, ITS-1 sequence, prevalence, Greece

## Abstract

This study presents the first molecular investigation into the prevalence and risk factors of *Eimeria* spp. infection among weaned dairy calves in Thessaly, Greece. Utilizing a cross-sectional design, 665 fecal samples were collected from 35 intensive dairy cattle farms and analyzed via genus-specific PCR and species-specific multiplex PCR targeting the internal transcribed spacer 1 (ITS-1) region. The overall molecular prevalence was found to be 46.3%, with *Eimeria bovis* (24.7%) and *Eimeria zuernii* (14.0%) emerging as the most prevalent species. Mixed infections were common, occurring in 51.0% of the positive cases. Multivariable analysis revealed that dairy calves aged less than 60 days had 2.15 times higher odds of infection compared to older calves. Environmental factors also significantly influenced infection rates, specifically ground flooring, the use of immovable/concrete water troughs and infrequent cleaning of floors, feeders and water troughs. These results highlight the high burden of pathogenic *Eimeria* in dairy cattle herds of Thessaly, Greece, and underscore the importance of integrating rigorous hygiene protocols with age-targeted management to control bovine coccidiosis.

## 1. Introduction

Bovine coccidiosis is a widespread parasitic disease of cattle caused by protozoa of the genus *Eimeria*. The infection represents a significant constraint to cattle health and productivity worldwide, leading to considerable economic losses in both dairy and beef production systems [1]. Although the financial impact of bovine coccidiosis is not always systematically quantified, available reports indicate substantial economic impacts, including production losses and the costs associated with prevention and treatment in countries such as Mexico [2], the United States [3] and Canada [4]. These losses are mainly attributed to reduced weight gain, impaired feed efficiency, decreased milk production, increased veterinary expenses and mortality in severe cases [1].

Across Europe and neighboring regions, *Eimeria* spp. infections in cattle are common, with reported prevalence varying considerably depending on geographic location, management practices and diagnostic methods employed [1,5,6]. Studies from various countries have documented moderate to high prevalence rates, particularly in intensive or semi-intensive management systems [5,7,8]. Young animals, especially calves under one year of age, are consistently reported as the most susceptible group, often exhibiting higher infection rates than adult cattle. Clinical manifestations range from subclinical infections to clinical disease, characterized by diarrhea (which may be hemorrhagic in severe cases), dehydration, anorexia, reduced growth performance, anemia and general weakness. Severe outbreaks are most frequently observed in calves, where high parasite burdens can lead to significant morbidity [5,7,8].

Environmental and management-related factors play a crucial role in the epidemiology of bovine coccidiosis. High stocking density, inadequate sanitation, accumulation of fecal material, and humid conditions favor oocyst survival and transmission [1,5]. In Mediterranean countries such as Greece, climatic conditions, particularly periods of increased rainfall and humidity, combined with certain housing and applied practices, may contribute to the persistence and spread of *Eimeria* infections in dairy cattle herds [8].

To date, at least 13 *Eimeria* species have been described in cattle. Among them, *Eimeria bovis* and *E. zuernii* are recognized as the most pathogenic species and are frequently associated with clinical coccidiosis and severe intestinal lesions in calves [3,8]. Other species, including *E. alabamensis*, *E. auburnensis*, *E. cylindrica* and *E. ellipsoidalis*, are also commonly detected but are generally considered less pathogenic [3,7].

Conventional diagnosis of bovine coccidiosis relies on microscopic identification of oocysts in fecal samples [1,3]. However, morphological similarities and overlapping dimensions among *Eimeria* species can complicate accurate differentiation and species-level identification. In this context, molecular diagnostic approaches, particularly polymerase chain reaction (PCR)-based assays, have emerged as reliable tools for sensitive and specific detection of *Eimeria* DNA [9]. Multiplex PCR techniques enable the simultaneous identification of multiple *Eimeria* species in a single reaction and are increasingly applied in epidemiological investigations [10]. In addition to its epidemiological relevance, the molecular characterization of *Eimeria* spp. provides important insights into parasite genetic diversity, population structure and host–parasite interactions. The internal transcribed spacer 1 (ITS-1) region, widely used for species differentiation, reflects genetic variability that may be associated with differences in pathogenicity, replication dynamics and adaptation to environmental conditions. Therefore, molecular identification of *Eimeria* spp. is not only a diagnostic tool but also contributes to a better understanding of the biological and regulatory mechanisms underlying infection dynamics in cattle. These molecular differences may influence key biological processes, including parasite replication, host cell invasion and species-specific pathogenicity.

Despite the recognized importance of bovine coccidiosis, molecular epidemiological data on the prevalence, species distribution and associated risk factors of *Eimeria* spp. in weaned dairy calves in Greece remain limited. Although molecular identification of *Eimeria* spp. has been reported in other regions worldwide, such integrated data combining species-level detection and risk factor analysis are currently lacking for Greek dairy production systems. Comprehensive epidemiological information is essential for the development of targeted control strategies, optimization of management practices, and reduction in unnecessary or indiscriminate use of anticoccidial drugs [3]. Therefore, the present study aimed to investigate the molecular prevalence and species distribution of *Eimeria* spp., focusing on ITS-1, in weaned dairy calves in Thessaly, Greece, and to evaluate animal- and farm-level risk factors associated with infection under intensive Mediterranean dairy production conditions.

## 2. Results

### 2.1. Prevalence of Eimeria Infection at the Genus Level

**Figure 1:**
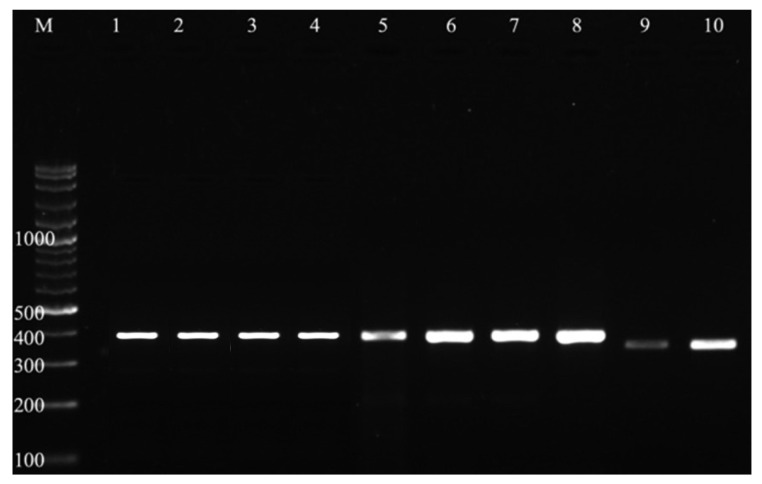
Agarose gel electrophoresis of *Eimeria*-positive samples at genus level. M: molecular weight marker, 1–10: *Eimeria*-positive samples from 348 to 546 bp, bp: base pairs.

Out of the 665 fecal samples analyzed, 308 (46.3%) tested positive for *Eimeria* spp. by conventional PCR. All samples were screened for *Eimeria* infection based on the presence of amplified fragments ranging from 348 to 546 bp observed during agarose gel electrophoresis (Figure 1). Table 1 presents, in detail, the prevalence rates of *Eimeria* spp. infection in weaned dairy calves among four prefectures of Thessaly, Greece.

### 2.2. Prevalence of Eimeria Infection at the Species Level

Out of the 308 examined calves, 151 (49.0%) presented with single *Eimeria* spp. infections, whereas 157 (51.0%) had mixed infections (Table 2). Among the single infections, *E. bovis* was the most prevalent species, detected in 76 calves (24.7%), followed by *E. zuernii* in 43 calves (14.0%) and *E. alabamensis* in 17 calves (5.8%). Lower prevalence rates were observed for *E. ellipsoidalis* (3.6%) and *E. cylindrica* (1.0%), while *E. auburnensis* was not detected in any of the examined samples (0.0%).

**Figure 2:**
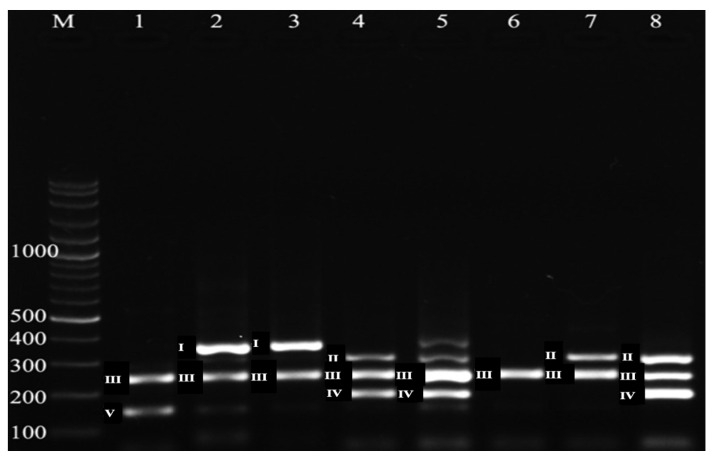
Agarose gel electrophoresis of *Eimeria*-positive samples at species level. M: molecular weight marker, 1–8: *Eimeria*-positive samples at different base pairs, I: *Eimeria zuernii* (350 bp), II: *Eimeria cylindrica* (304 bp), III: *Eimeria bovis* (238 bp), IV: *Eimeria alabamensis* (184 bp), and V: *Eimeria ellipsoidalis* (148 bp), bp: base pairs.

Mixed *Eimeria* spp. infections were slightly more common than single infections, accounting for 157 (51.0%) cases out of 308 examined calves. The detailed distribution of species combinations in mixed infections is presented in Table 2. Moreover, all samples were screened for *Eimeria* species infection based on the presence of different amplified fragments per species observed during agarose gel electrophoresis (Figure 2).

Analysis of mixed infections (*n* = 157) revealed that *E. bovis* and *E. zuernii* were the most frequently detected species, present in 64.9% and 63.7% of mixed infections, respectively, followed by *E. alabamensis*, *E. ellipsoidalis* and *E. cylindrica* (Table 2).

### 2.3. Descriptive Findings of the Examined Risk Factors

Among the 665 weaned dairy calves included in the analysis, the prevalence of *Eimeria* spp. infection differed across animal- and farm-related variables (Table 3). Calves aged ≤ 60 days presented a prevalence of 49.7%, whereas calves older than 60 days showed a prevalence of 42.5%. Infection prevalence was 49.2% in calves housed on ground floors and 43.9% in those housed on concrete floors. Regarding water trough type, prevalences of 58.1% and 38.5% were observed in calves using immovable and movable troughs, respectively. Concerning hygiene practices, calves from pens where floors were cleaned less frequently than once per month had a prevalence of 69.8%, compared to 30.3% in pens cleaned monthly. Prevalence was 50.6% when feeders were cleaned weekly and 41.8% when cleaned daily, while corresponding prevalences for water trough cleaning were 48.9% and 46.7%, respectively.

### 2.4. Risk Factors of Dairy Calves Infected by Eimeria spp.

The associations between selected animal- and farm-level factors and *Eimeria* spp. infection in weaned dairy calves are presented in Table 3. Age was significantly associated with infection status (*p* ≤ 0.001). Calves aged ≤60 days exhibited a 2.15-fold higher odds of *Eimeria* spp. infection compared to calves >60 days of age (95% CI 1.453 to 3.583, *p* ≤ 0.001). Housing conditions were also significantly related to infection prevalence. Calves housed on ground floors showed 1.89-fold higher odds of infection compared to those kept on concrete floors (95% CI 1.112 to 2.523, *p* ≤ 0.05). The water trough type demonstrated a strong association with infection status. Calves with access to immovable/concrete water troughs had significantly increased odds of infection compared to those using movable troughs. (95% CI 1.993–3.875, *p* ≤ 0.05).

Farm-related variables were also significantly associated with *Eimeria* spp. positivity. Less frequent cleaning of floors (i.e., longer than once per month) was strongly associated with infection (95% CI 4.932–7.685, *p* ≤ 0.001). Similarly, cleaning feeders once per week, compared to daily cleaning, significantly increased the odds of infection (95% CI 1.283–2.984, *p* ≤ 0.05). Finally, infrequent cleaning of water troughs (i.e., longer than once per week) exhibited a 1.13-fold higher odds of *Eimeria* spp. infection compared to cleaning of water troughs once per week (95% CI 1.027–1.233, *p* ≤ 0.05).

No statistically significant associations were observed for the remaining evaluated variables (*p* > 0.05). Specifically, sex, vaccination status, herd size and type of feeder were not identified as significant predictors of *Eimeria* spp. infection in the studied dairy calves.

## 3. Discussion

The objective of the present study was to determine the molecular prevalence of *Eimeria* spp. in weaned dairy calves in Thessaly, Greece, and to investigate potential risk factors associated with infection at both the animal and farm levels.

Bovine coccidiosis is widely distributed in the dairy production industry worldwide and remains a common protozoan infection of calves [1]. In the present study, the overall molecular prevalence of *Eimeria* spp. was 46.3% in weaned dairy calves in Thessaly, Greece. This prevalence is comparable to reports from other countries conducted in Southeast Asia, where prevalence estimates of 48% in Thailand [11], 53% in Indonesia [12], 56% in Malaysia [5] and 47% in China [13] have been documented. Variations among studies may reflect differences in the age groups examined, climatic conditions, diagnostic approaches and farm management practices [14,15]. Given that *Eimeria* infection predominantly affects young animals, particularly around the weaning period, the relatively high prevalence observed in this study is consistent with the susceptibility of calves at this critical stage of development. Additionally, intensive production systems and hygiene-related factors, such as floor cleaning frequency, feeder and water trough sanitation, type of flooring and water supply system, may further influence exposure and transmission dynamics within dairy cattle herds [5,7,8]. These findings should also be interpreted in the context of existing molecular epidemiological studies worldwide.

Molecular detection of *Eimeria* spp. in cattle has been increasingly applied in recent years, with studies from various regions reporting comparable prevalence rates and species distributions. In this context, the findings of the present study are consistent with existing literature, particularly regarding the predominance of *E. bovis* and *E. zuernii* and the frequent occurrence of mixed infections. Nevertheless, the present study provides novel epidemiological data for Greece, where molecular-based investigations in dairy calves remain scarce, and further contributes by integrating species-level identification with a detailed assessment of management-related risk factors under intensive Mediterranean production conditions.

Mixed *Eimeria* infections were commonly identified in the present study, a finding that aligns with previous molecular investigations conducted in different geographical regions [13,16]. Five *Eimeria* species were detected, namely *E. bovis*, *E. zuernii*, *E. ellipsoidalis*, *E. alabamensis* and *E. cylindrica*, indicating a considerable diversity of circulating species in weaned dairy calves in Thessaly. The broader spectrum of species identified in this study compared to earlier reports relying solely on coprological flotation methods may be attributed to the higher analytical sensitivity and specificity of PCR-based assays, which allow accurate differentiation of morphologically similar oocysts [3,17]. Despite the detection of multiple species, *E. bovis* and *E. zuernii* were the predominant species, corroborating their recognized epidemiological importance and pathogenic potential in young calves. The predominance of these two species has been consistently reported in molecular studies from various countries [8,12,13,14,16,18,19], supporting their global distribution and major role in bovine coccidiosis. This predominance may reflect species-specific differences in replication strategies, host cell invasion efficiency and molecular adaptation mechanisms, which have been suggested to influence their higher pathogenic potential.

Beyond species identification, the molecular detection of *Eimeria* spp. based on the ITS-1 region provides indirect insight into parasite genetic diversity and population dynamics within dairy production systems. The high frequency of mixed infections observed in the present study suggests co-circulation of multiple genetically distinct species within the same host, which may influence parasite replication dynamics, host cell invasion processes and interspecific interactions at the molecular level within the intestinal environment. Although the present study did not investigate gene expression or functional genomics, the molecular patterns identified contribute to the understanding of how genetically distinct *Eimeria* spp. coexist and interact under field conditions. Therefore, the molecular identification of *Eimeria* spp. in the present study provides a framework for understanding how genetic variability among species may be linked to regulatory mechanisms governing infection establishment and disease progression.

The multivariable analysis identified age as a significant factor associated with *Eimeria* spp. infection. Calves aged ≤ 60 days exhibited higher odds of infection compared to older calves, highlighting the increased susceptibility of animals during the early postnatal and weaning period. This finding is consistent with numerous epidemiological studies demonstrating that *Eimeria* infections predominantly affect young calves, in which clinical and subclinical coccidiosis are more frequently observed than in older cattle [19,20,21,22]. The increased vulnerability of young animals is generally attributed to their immature immune system and limited prior exposure to *Eimeria* oocysts [3]. In contrast, older cattle tend to develop partial protective immunity following repeated exposure under field conditions, which reduces the likelihood of clinical disease and lowers oocyst shedding [23]. Given the recognized economic impact of coccidiosis in young calves [1], particularly around weaning, the age-related pattern observed in the present study further supports the importance of targeted control measures during this critical production stage. Nevertheless, some studies have reported no significant age-related differences in infection rates, suggesting that management practices, environmental contamination levels and herd immunity dynamics may also influence age-associated risk patterns [24,25,26].

A significant association was also identified between *Eimeria* infection and housing floor type. Calves housed on ground floors exhibited higher odds of infection compared to those maintained on concrete flooring. This observation may be explained by differences in hygiene management, as concrete surfaces are generally easier to clean and disinfect, thereby reducing environmental accumulation and sporulation of *Eimeria* oocysts. Similar associations between non-cemented flooring and increased coccidial infection risk have been reported in previous epidemiological studies [3,20], supporting the role of housing infrastructure in pathogen transmission dynamics.

The water supply system was another significant factor associated with *Eimeria* infection status. Calves provided with water through immovable or concrete troughs showed higher odds of infection compared to those using movable trough systems. Fixed water troughs may facilitate the persistence and buildup of organic material and fecal contamination when not adequately cleaned, increasing the likelihood of oocyst ingestion [20].

In addition, hygiene-related management practices were significantly associated with infection risk [3,27]. Calves from farms where feeder boxes were cleaned weekly had higher odds of infection compared to farms implementing daily cleaning routines. Likewise, reduced frequency of floor cleaning was strongly associated with increased infection risk. Finally, a tendency toward higher *Eimeria* prevalence was observed in calves from farms where water troughs were cleaned less frequently, suggesting that suboptimal sanitation of drinking equipment may contribute to increased environmental contamination and parasite transmission. These findings reinforce the importance of hygiene measures in limiting environmental contamination with infective oocysts. Inadequate cleaning of housing areas, feeding equipment and water sources may promote oocyst survival and enhance transmission within calf-rearing units [3,22,27].

Overall, the findings of the present study highlight that both animal- and farm-related factors contribute to the epidemiology of *Eimeria* infection in weaned dairy calves. The increased susceptibility observed in younger calves underscores the importance of targeted preventive strategies during early life and the weaning period, when animals are immunologically immature and more vulnerable to environmental challenges. At the same time, the strong associations identified with housing floor type, water trough system, and cleaning frequency emphasize the critical role of hygiene and environmental management in limiting oocyst accumulation and transmission. Implementation of regular and thorough cleaning protocols, use of easily washable flooring materials, and proper maintenance of feeding and watering equipment may substantially reduce infection pressure within calf-rearing units. Therefore, integrated control programs combining age-targeted monitoring with improved hygiene practices are essential to mitigate the impact of bovine coccidiosis in intensively managed dairy farms.

## 4. Materials and Methods

### 4.1. Herd History

The study was conducted on 35 commercial dairy cattle farms located in the region of Thessaly, Greece. The farms were selected to be representative of intensive dairy production in Thessaly, based on accessibility, herd size and willingness to participate, covering a range of management practices typical of the region. Thessaly was strategically selected for the present investigation not only because it constitutes one of the most intensive dairy-producing regions of Greece, but also due to the unique environmental and epidemiological pressures that may influence the transmission dynamics of *Eimeria* spp. The region has recently experienced severe flooding events that caused prolonged water stagnation, redistribution of fecal material and structural damage to livestock facilities, conditions that favor oocyst survival, sporulation and environmental dissemination. In parallel, Thessaly is characterized by recurrent heat waves and prolonged periods of high ambient temperatures, which may compromise calf immune competence through heat stress while simultaneously modifying management practices (e.g., increased congregation around water sources), thereby facilitating fecal-oral transmission. Furthermore, post-flood herd restructuring and the importation or movement of replacement animals from different geographical origins may introduce new *Eimeria* species or strains, potentially increasing infection pressure and the occurrence of mixed infections. The convergence of climatic extremes, environmental disruption and livestock movement creates a dynamic epidemiological setting that may intensify exposure of immunologically immature calves during the critical weaning period, rendering Thessaly a particularly relevant region for a molecular investigation of *Eimeria* infection and associated risk factors.

All farms operated under an intensive management system and reared Holstein cattle. The mean number of lactating cows per farm was approximately 220 (±50), representing typical medium- to large-scale intensive dairy farms in the region [28,29]. Calves were separated from their dams shortly after birth and housed individually in hutches or individual pens until weaning. They were fed colostrum within the first hours of life according to standard farm protocols, followed by whole milk or milk replacer offered two times daily. Calf starter concentrate and fresh water were provided ad libitum from the first week of life. Weaning generally occurred between 8 and 10 weeks of age, depending on body weight and starter intake. During the weaning period, calves were gradually transitioned from a milk-based diet to solid feed and subsequently moved to group pens. Hygiene practices, bedding management and stocking density varied slightly among farms but followed routine farm management practices commonly applied in medium- to large-scale intensive dairy cattle farms in Greece.

Preventive herd health management followed routine vaccination programs implemented by the attending veterinarians. Vaccination protocols commonly included immunization of pregnant heifers and cows during the dry period (approximately one month before calving) against neonatal calf diarrhea pathogens (rotavirus, coronavirus, and *Escherichia coli* K99) using Rotavec Corona^®^ (MSD Animal Health, Boxmeer, The Netherlands). Vaccination against clostridial diseases (Bravoxin, MSD Animal Health, Boxmeer, The Netherlands) was also routinely applied. In addition, all farms implemented biannual vaccination programs against bovine viral diarrhea (BVD) and infectious bovine rhinotracheitis (IBR), in accordance with national veterinary guidelines (Bovilis^®^, MSD Animal Health, Boxmeer, The Netherlands).

In addition, it should be considered that dairy cattle herds may act as continuous reservoirs of *Eimeria* spp., as adult cattle can shed low numbers of oocysts without showing clinical signs [1]. Periods of physiological stress, particularly around calving, may be associated with increased oocyst excretion, contributing to contamination of maternity pens and surrounding areas. Consequently, newborn calves may be exposed to infective oocysts early in life [5,7,8]. Although calves in the present study were initially housed in individual hutches or pens, suboptimal hygiene practices, delayed bedding replacement and accumulation of fecal material could facilitate oocyst survival and sporulation, thereby maintaining environmental infection pressure within calf-rearing units [6,7].

### 4.2. Experimental Design and Sample Collection

A cross-sectional study was conducted between May 2024 and July 2025 in 35 commercial dairy farms. The target population consisted of dairy calves on weaning day, with ages ranging from 51 to 75 days. For analysis, calves were grouped as ≤60 days (51–60 days) and >60 days (64–75 days). A total of 665 fecal samples were collected, with 19 samples obtained from each farm, sufficient for robust prevalence estimation and evaluation of risk factors. Of the 665 weaned dairy calves sampled, 164 were male and 501 were female. Calves were sampled on the day of weaning to assess *Eimeria* spp. infection status during the critical transition from milk to solid feed. The weaning stage was selected due to the increased susceptibility of calves to enteric infections associated with dietary, environmental and social stressors occurring at this time.

Calves were selected randomly, using a random number generator to ensure equal probability of inclusion, from those scheduled for weaning on each farm during the study period. Fresh fecal samples were collected directly from the rectum using disposable gloves to avoid environmental contamination. Each sample was placed in a sterile plastic container, properly labeled with farm identification and animal code, and transported under refrigerated conditions (+4 °C) to the Laboratory of Parasitology and Parasitic Diseases, Veterinary School of the Aristotle University of Thessaloniki, for further parasitological and molecular analyses. All sampling procedures were performed in accordance with standard animal welfare guidelines and with the consent of the farm owners.

### 4.3. DNA Extraction from Fecal Samples

Genomic DNA was extracted from all collected fecal samples using the QIAamp^®^ Fast DNA Stool Mini Kit (Qiagen, Hilden, Germany), following the manufacturer’s instructions for pathogen DNA isolation from stool specimens.

Prior to extraction, samples were subjected to an additional mechanical–thermal disruption step to enhance oocyst wall rupture and improve DNA yield. Specifically, three freeze–thaw cycles were applied, consisting of incubation at 80 °C in a water bath for 5 min, followed by rapid freezing at −20 °C for 5 min. The 5 min freezing step refers to the incubation period at −20 °C in a standard laboratory freezer following the 80 °C water bath; temperature equilibration occurs gradually during this period. This procedure was incorporated to facilitate effective lysis of *Eimeria* oocysts, which possess a resistant wall structure.

Following the extraction protocol, DNA was eluted in 50 μL of the provided elution buffer. The purified DNA samples were stored at −20 °C until further molecular analysis.

### 4.4. Amplification of ITS-1 Region for Genus-Level Identification

All extracted DNA samples obtained from the 665 fecal specimens were initially screened using a genus-specific PCR assay for the detection of *Eimeria* spp. Forward and reverse primers, described by Al-Jubory and Al-Rubaie [14] and produced by Eurofins Genomics GmbH (Ebersberg, Germany), targeting the ITS-1 region of the 18S rRNA gene were used (Table 4). This region allows reliable molecular detection of *Eimeria* spp. at the genus level.

PCRs were performed in a final volume of 20 μL, containing 10 μL of commercial PCR master mix, 0.25 μL of each primer (total primer volume 0.5 μL), 0.5 μL of template DNA, and nuclease-free water to reach the final volume. Amplification was carried out under the following thermal cycling conditions: initial denaturation at 95 °C for 5 min, followed by 30 cycles of denaturation at 95 °C for 30 s, annealing at 58 °C for 30 s, and extension at 72 °C for 45 s, with a final extension step at 72 °C for 5 min.

PCR products were separated on 1% agarose gel stained with ethidium bromide and visualized under ultraviolet illumination (Super-Bright UV-Pad Edge 26MX, VWR International Ltd., UK). Amplicons ranging from 348 to 546 bp were considered positive for *Eimeria* spp.

Only samples that tested positive in the genus-specific PCR assay were subsequently subjected to multiplex PCR analysis for species-level identification.

### 4.5. Multiplex PCR for Species-Level Identification

Samples that tested positive in the genus-specific PCR assay were further analyzed using species-specific multiplex PCR to identify individual *Eimeria* spp.

Forward and reverse primers, described by Al-Jubory and Al-Rubaie [14] and produced by Eurofins Genomics GmbH (Ebersberg, Germany), targeting each species are listed in Table 5. Each multiplex PCR was performed in a final volume of 20 μL, consisting of 10 μL of PCR master mix, 6.5 μL of nuclease-free water, 0.25 μL of each forward primer (total 1.5 μL), 0.25 μL of each reverse primer (total 1.5 μL) and 0.5 μL of the genus-positive PCR product as the DNA template. The thermal cycling conditions were the same as those used for the initial genus-specific PCR.

Five microliters of each multiplex PCR product were separated by electrophoresis on 1% agarose gel alongside a 100 bp DNA ladder and run for 30 min. Gels were stained with ethidium bromide and visualized under ultraviolet transillumination (Super-Bright UV-Pad Edge 26MX, VWR International Ltd., Leicestershire, UK). Bands corresponding to the expected amplicon sizes for each *Eimeria* species were recorded for species-level identification.

### 4.6. Data Collection and Risk Factor Assessment

Structured questionnaires were developed to identify potential risk factors associated with *Eimeria* spp. infection in weaned dairy calves. The questionnaire collected information at both the farm and individual animal levels. At the farm level, data included herd size, type of floor, type of feeders and water troughs and cleaning frequency of feeders, water troughs and floors. At the individual animal level, information was obtained on age, sex and vaccination status. Categories used in the analysis reflect the reported routine practices on each farm.

All information was collected through direct interviews with farm owners or managers and cross-checked with farm records when available. These data were subsequently used to assess associations between management practices, calf characteristics and the presence of *Eimeria* infection.

### 4.7. Data Handling—Statistical Analyses

Prevalence rate values and their 95% confidence intervals were estimated using the Epitools (https://epitools.ausvet.com.au/ciproportion, accessed on 21 March 2026) and the Wilson score interval method [30]. Associations between potential risk factors and *Eimeria* infection status in weaned calves were evaluated using a binary logistic regression model [31]. The dependent variable (Y) was the infection status of each calf (0 = negative, 1 = positive for *Eimeria* spp.). Independent variables (X) included both animal- and farm-level factors:

- Age class of the calf (X_1_, 2 levels: 0 = ≤60 days, 1 = >60 days),
- Sex (X_2_, 2 levels: 0 = male, 1 = female),
- Vaccination status (X_3_, 2 levels: 0 = unvaccinated, 1 = vaccinated),
- Herd size (X_4_, 2 levels: 0 = <100 cows, 1 = ≥100 cows),
- Type of floor (X_5_, 2 levels: 0 = concrete, 1 = ground),
- Type of feeder (X_6_, 2 levels: 0 = movable, 1 = immovable/concrete),
- Type of water trough (X_7_, 2 levels: 0 = movable, 1 = immovable/concrete),
- Cleaning frequency of floor (X_8_, 2 levels: 0 = once a month, 1 = longer than a month),
- Cleaning frequency of feeders (X_9_, 2 levels: 0 = every day, 1 = once a week), and
- Cleaning frequency of water troughs (X_10_, 2 levels: 0 = once a week, 1 = longer than a week).

The logistic regression model was expressed as:

$$Y=\alpha +\beta_{1}X_{1}+\beta_{2}X_{2}+\beta_{3}X_{3}+\beta_{4}X_{4}+\beta_{5}X_{5}+\beta_{6}X_{6}+\beta_{7}X_{7}+\beta_{8}X_{8}+\beta_{9}X_{9}+\beta_{10}X_{10}$$

where β_1_–β_10_ represent the regression coefficients of the respective predictors.

The Wald χ^2^ test was used to evaluate the statistical significance of each individual predictor. Model fit was assessed using the Hosmer–Lemeshow goodness-of-fit test, along with the Cox and Snell R^2^ and Nagelkerke R^2^ indices [31]. Statistical significance was set at *p* ≤ 0.05. All analyses were performed using SPSS version 28.0 (IBM Corp., Armonk, NY, USA).

## 5. Conclusions

This study provides the first molecular epidemiological data on *Eimeria* spp. infection in weaned dairy calves in Thessaly, Greece. It revealed a high prevalence of infection (46.3%) among weaned dairy calves, with *E. bovis* and *E. zuernii* identified as the most frequent and pathogenic species. The study demonstrated that calves were particularly vulnerable during the weaning period, with those aged 60 days or younger facing significantly higher odds of infection. Furthermore, environmental management and hygiene practices played a critical role in transmission. Factors such as the type of ground flooring, the use of immovable water troughs and infrequent cleaning of facilities (i.e., flooring, feeders and water troughs) significantly increase the risk of *Eimeria* spp. infection. Ultimately, these findings suggest that mitigating the economic and health impacts of bovine coccidiosis in intensive dairy cattle systems requires integrated control strategies that combine age-specific monitoring with rigorous sanitation protocols to limit *Eimeria* oocyst accumulation.

## Figures and Tables

**Table 1:** Prevalence rates (±95% confidence intervals) of *Eimeria* spp. infection in weaned dairy calves of four prefectures of Thessaly, Greece.

Region	Prefecture	Farms	*Eimeria*-Positive/Total Samples	Prevalence (%)	95% CI	
					Lower	Upper
Thessaly	Larissa	11	97/209	46.4	39.8	53.2
	Karditsa	8	80/152	52.6	44.7	60.4
	Trikala	8	65/152	42.7	35.2	50.7
	Magnesia	8	66/152	43.4	35.8	51.4
	Total	35	308/665	46.3	42.6	50.1

CI: confidence interval.

**Table 2:** Prevalence and 95% confidence interval of *Eimeria* spp. in the studied calves (*n* = 308).

Species	Number of Positive Fecal Samples	Prevalence (%)	95% CI	
			Lower	Upper
Single *Eimeria* spp. infection				
*E. bovis* (a)	76	24.7	20.2	29.8
*E. zuernii* (b)	43	14.0	10.5	18.3
*E. alabamensis* (c)	17	5.8	3.5	8.7
*E. ellipsoidalis* (d)	11	3.6	2.0	6.3
*E. cylindrica* (e)	3	1.0	0.3	2.8
*E. auburnensis* (f)	0	0.0	0.0	0.0
Total	151	49.0	43.5	54.6
Mixed *Eimeria* spp. infections				
(a) and (b)	25	8.1	5.6	11.7
(a) and (c)	24	7.8	5.3	11.3
(a) and (d)	21	6.8	4.5	10.2
(b) and (e)	17	5.5	3.5	8.7
(b) and (c)	15	4.9	3.0	7.9
(b) and (d)	15	4.9	3.0	7.9
(a), (c) and (d)	12	3.9	2.2	6.7
(a), (b) and (c)	8	2.6	1.3	5.0
(b), (c) and (e)	8	2.6	1.3	5.0
(a), (b) and (d)	5	1.6	0.7	3.7
(a), (b), (d) and (e)	5	1.6	0.7	3.7
(a), (b), (c), (d) and (e)	2	0.6	0.2	2.3
Total	157	51.0	45.4	56.5
*Eimeria* spp. in mixed infections				
(a)	102	64.9	57.2	71.9
(b)	100	63.7	55.9	70.8
(c)	69	43.9	36.4	51.7
(d)	60	38.2	30.9	46.0
(e)	32	20.4	14.8	27.3

CI: confidence interval.

**Table 3:** Prevalence (%), odds ratios with 95% confidence intervals, and *p*-values of the risk factors associated with *Eimeria* spp. infection in weaned dairy calves.

Factors	Number of Positive/Total Fecal Samples	Prevalence (%)	Odds Ratio	95% CI		*p*-Value
				Lower	Upper	
Age						
>60 days	133/313	42.5	1			
≤60 days	175/352	49.7	2.15	1.453	3.583	0.000
Type of floor						
Concrete	159/362	43.9	1			
Ground	149/303	49.2	1.89	1.112	2.523	0.032
Type of water trough						
Movable	154/400	38.5	1			
Immovable/Concrete	154/265	58.1	2.95	1.993	3.875	0.002
Cleaning frequency of floor						
Once a month	120/396	30.3	1			
Longer than a month	188/269	69.8	5.29	4.932	7.685	0.000
Cleaning frequency of feeders						
Every day	135/323	41.8	1			
Once a week	173/342	50.6	1.42	1.283	2.984	0.033
Cleaning frequency of water troughs						
Once a week	149/352	42.3	1			
Longer than a week	159/313	50.8	1.13	1.027	1.233	0.045

CI: confidence intervals.

**Table 4:** Primers and expected product size (bp) for *Eimeria* identification at genus level.

Primers		Product Size (bp)
Forward:	5′-GCAAAAGTCGTAACACGGTTTCCG-3′	348–546
Reverse:	5′-CTGCAATTCACAATGCGTATCGC-3′	

bp: base pairs.

**Table 5:** Primers and expected product size (bp) for *Eimeria* identification at species level.

*Eimeria* Species	Primers		Product Size (bp)
*Eimeria zuernii*	Forward:	5′–AACATGTTTCTACCCACTAC–3′	344
	Reverse:	5′–CGATAAGGAGGAGGACAAC–3′	
*Eimeria cylindrica*	Forward:	5′–GACATTTAAAAAACCGATTGGT–3′	304
	Reverse:	5′–GGCTGCAATAAGATAGACATA–3′	
*Eimeria auburnensis*	Forward:	5′–TAAATTGGTGCGATGAGGGA–3′	295
	Reverse:	5′–GCAATGAGAGAAAGATTTAATA–3′	
*Eimeria bovis*	Forward:	5′–TCATAAAACATCACCTCCAA–3′	238
	Reverse:	5′–ATAATTGCGATAAGGGAGACA–3′	
*Eimeria alabamensis*	Forward:	5′–CATTCACACATTGTTCTTTCAG–3′	184
	Reverse:	5′–GCTTCCAAACTAATGTTCTG–3′	
*Eimeria ellipsoidalis*	Forward:	5′–CAACGTTTTTCCTTTTCCTATCA–3′	148
	Reverse:	5′–ACTGCGATGAGAGAGAGCG–3′	

bp: base pairs.

## Data Availability

The original contributions presented in this study are included in the article. Further inquiries can be directed to the corresponding author.
